# Supplementary material for: Distinguishing Incubation and Acute Disease Stages of Mild-to-Moderate COVID-19
Source: Viruses. 2022 Jan 20;14(2):203. doi: 10.3390/v14020203 (PMC8875077; doi:10.3390/v14020203)
Supplement: Supplementary file 1 [file viruses-14-00203-s001.zip › viruses-1567416-supplementary.pdf]

Supplementary Material for

Distinguishing Incubation and Acute Disease Stages of Mild-to-Moderate COVID-19

Michael Müller, Johann Volzke *et al.*

Corresponding author. Email: Brigitte Müller-Hilke, brigitte.mueller-hilke@med.uni-rostock.de

The file includes:

Figure S1

Tables S1 to S17

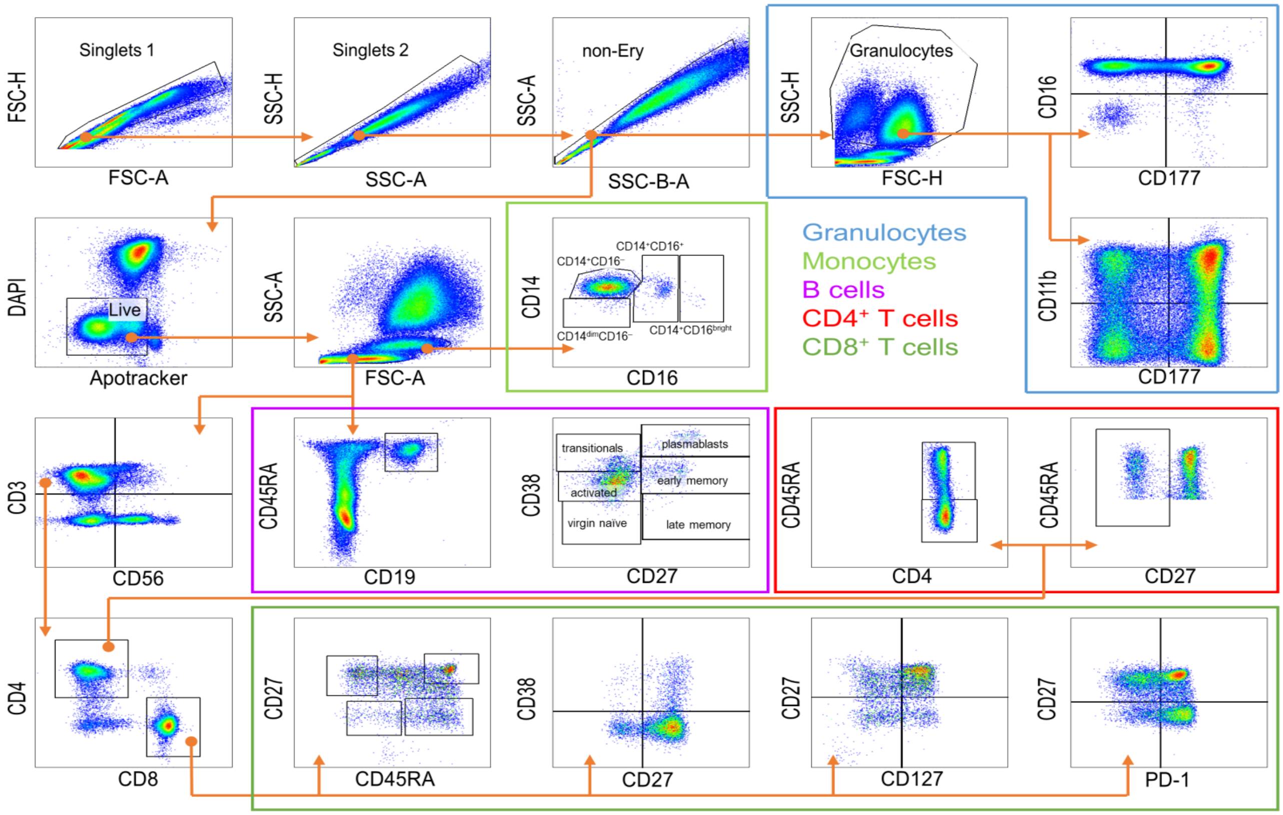

Table S1. CD14+ Multi-Pathway Signaling.

Protein [1/µg]	Controls (n = 12) Median (IQR)	COVID-19 (n = 11) Median (IQR)	MWW p-value	p-Protein/Protein	Controls (n = 12) Median (IQR)	COVID-19 (n = 11) Median (IQR)	MWW p-value
CREB	35.8 (26.0 – 53.6)	8.0 (3.87 – 36.3)	0.0792	p-CREB/CREB	0.0013 (0.0006 – 0.0108)	0.0108 (0.0019 – 0.0340)	0.212
JNK	15.0 (12.5 – 23.3)	10.0 (6.89 – 13.6)	0.051	p-JNK/JNK	0.0294 (0.0113 – 0.0621)	0.0483 (0.0259 – 0.0670)	0.566
NF-κB	0.10 (0.10 – 0.20)	0.09 (0.06 – 0.15)	0.288	p-NF-κB/NF-κB	1.9189 (0.0412 – 7.6768)	2.4977 (1.7329 – 5.5697)	0.695
p38	3.74 (3.32 – 10.9)	2.21 (1.47 – 12.2)	0.091	p-p38/p38	0.0193 (0.0075 – 0.0730)	0.1469 (0.0696 – 0.1703)	0.060
ERK1/2	0.18 (0.14 – 0.21)	0.14 (0.08 – 0.20)	0.260	p-ERK1/2/ERK1/2	0.0145 (0.0008 – 0.1724)	0.1881 (0.0683 – 0.2638)	0.151
Akt	13.8 (11.2 – 16.3)	8.57 (5.90 – 16.3)	0.260	p-Akt/Akt	0.0107 (0.0048 – 0.0166)	0.0099 (0.0071 – 0.0113)	0.786
p70S6k	27.1 (20.7 – 29.3)	9.97 (5.72 – 15.6)	**0.0036**	p-p70S6K/p70S6k	0.3323 (0.1159 – 0.5749)	0.4024 (0.1059 – 0.7781)	0.786
STAT3	157 (112 – 240)	58.9 (33.8 – 137)	**0.023**	p-STAT3/STAT3	0.0429 (0.0217 – 0.1226)	0.1209 (0.0274 – 0.5245)	0.316
STAT5	33.2 (28.4 – 51.7)	27.4 (15.5 – 54.5)	0.449	p-STAT5/STAT5	0.0444 (0.0107 – 0.0580)	0.0357 (0.0092 – 0.0882)	0.740

MWW: Mann-Whitney-Wilcoxon test.

Table S2. CD3⁺ Multi-Pathway Signaling.

	Controls (n =10)	COVID-19 (n = 14)	MWW		Controls (n = 10)	COVID-19 (n = 14)	MWW
Protein [1/μg]	Median (IQR)	Median (IQR)	p-value	p-Protein/Protein	Median (IQR)	Median (IQR)	p-value
CREB	210 (131 – 347)	242 (61.4 – 412)	0.977	p-CREB/CREB	1.576 (0.738 – 4.597)	1.428 (0.023 – 29.87)	0.886
JNK	396 (321 – 609)	332 (174 – 528)	0.667	p-JNK/JNK	0.098 (0.036 – 0.353)	0.130 (0.043 – 0.174)	0.886
NF-κB	95.2 (63.8 – 110)	44.7 (1.13 – 108)	0.341	p-NF-κB/NF-κB	3.024 (0.352 – 20.42)	4.258 (3.591 – 6.356)	0.886
p38	144 (120 – 180)	146 (47.4 – 183)	0.796	p-p38/p38	0.983 (0.322 – 8.661)	0.850 (0.207 – 3.785)	0.508
ERK1/2	136 (95.8 – 182)	85.4 (1.93 – 156)	0.235	p-ERK1/2/ERK1/2	0.034 (0.002 – 0.283)	0.125 (0.074 – 0.354)	0.437
Akt	5461 (2263 – 10271)	697 (33 – 6049)	0.096	p-Akt/Akt	0.611 (0.092 – 3.065)	0.023 (0.008 – 0.149)	**0.036**
p70S6k	202 (150 – 257)	183 (101 – 257)	0.709	p-p70S6K/p70S6k	0.251 (0.105 – 0.566)	0.350 (0.096 – 0.648)	0.752
STAT3	42640 (17715 – 62047)	3656 (690 – 26623)	**0.013**	p-STAT3/STAT3	0.024 (0.004 – 0.110)	0.107 (0.041 – 0.340)	0.138
STAT5	7070 (4494 – 10922)	630 (106 – 3715)	**0.011**	p-STAT5/STAT5	0.044 (0.009 – 0.056)	0.077 (0.016 – 0.206)	0.172

Table S3. CD19+ Multi-Pathway Signaling.

	Controls (n = 12)	COVID-19 (n = 11)	MWW		Controls (n = 12)	COVID-19 (n = 11)	MWW
Protein [1/μg]	Median (IQR)	Median (IQR)	p-value	p-Protein/Protein	Median (IQR)	Median (IQR)	p-value
CREB	309 (144 – 576)	216 (6.30 – 642)	0.950	p-CREB/CREB	0.0615 (0.0228 – 1.6268)	0.0647 (0.0038 – 10.281)	0.950
JNK	120 (66.6 – 273)	59.3 (19.7 – 158)	0.345	p-JNK/JNK	0.0519 (0.0221 – 0.1445)	0.0231 (0.0041 – 0.1370)	0.414
NF-κB	9.51 (1.02 – 64.2)	22.2 (0.31 – 56.0)	0.662	p-NF-κB/NF-κB	2.1005 (1.3766 – 2.8504)	1.4228 (0.0477 – 3.4136)	0.950
p38	117 (56 – 174)	43.7 (7.04 – 149)	0.491	p-p38/p38	0.2374 (0.0764 – 2.7023)	0.0956 (0.0159 – 2.2763)	0.573
ERK1/2	25.7 (1.19 – 162)	20.8 (0.20 – 68.4)	0.345	p-ERK1/2/ERK1/2	0.0982 (0.0342 – 0.2211)	0.0635 (0.0019 – 0.1417)	0.491
Akt	44.7 (32.6 – 103)	57.7 (24.9 – 67.5)	0.950	p-Akt/Akt	0.0159 (0.0090 – 0.0342)	0.0096 (0.0024 – 0.0150)	0.282
p70S6k	73.1 (62.1 – 92.5)	69.1 (25.1 – 123)	0.852	p-p70S6K/p70S6k	0.2391 (0.1140 – 0.7140)	0.4369 (0.0945 – 1.0544)	1.00
STAT3	437 (169 – 1249)	154 (32.5 – 653)	0.282	p-STAT3/STAT3	0.0551 (0.0431 – 0.3685)	0.0305 (0.0052 – 0.1297)	0.414
STAT5	124 (19.6 – 281)	46.8 (22.4 – 109)	0.414	p-STAT5/STAT5	0.0449 (0.0370 – 0.0745)	0.0148 (0.0035 – 0.0278)	0.059

Table S4. CD11b⁺ Multi-Pathway Signaling.

	Controls (n = 11)	COVID-19 (n = 9)	MWW		Controls (n = 11)	COVID-19 (n = 9)	MWW
Protein [1/μg]	Median (IQR)	Median (IQR)	p-value	p-Protein/Protein	Median (IQR)	Median (IQR)	p-value
CREB	1.40 (1.31 – 2.24)	2.28 (0.69 – 2.87)	0.766	p-CREB/CREB	0.0005 (0.0003 – 0.0114)	0.0009 (0.0001 – 0.0345)	0.456
JNK	12.8 (11.9 – 24.7)	14.2 (7.40 – 21.2)	0.552	p-JNK/JNK	0.0058 (0.0021 – 0.0362)	0.0475 (0.0210 – 0.0494)	0.112
NF-κB	0.33 (0.29 – 0.47)	0.22 (0.14 – 0.56)	0.456	p-NF-κB/NF-κB	0.0746 (0.0106 – 2.2910)	2.0574 (0.0322 – 4.547)	0.331
p38	2.76 (1.96 – 6.34)	3.52 (1.09 – 6.75)	0.552	p-p38/p38	0.0138 (0.0033 – 0.0930)	0.0737 (0.0069 – 0.1237)	0.370
ERK1/2	0.11 (0.07 – 0.25)	0.13 (0.04 – 0.20)	0.412	p-ERK1/2/ERK1/2	0.0022 (0.0002 – 0.1471)	0.0793 (0.0007 – 0.1605)	0.552
Akt	13.1 (10.5 – 26.0)	18.0 (5.71 – 25.9)	0.603	p-Akt/Akt	0.0050 (2.1×10^{-5} – 0.0077)	0.0051 (0.0038 – 0.0099)	0.456
p70S6k	4.74 (2.89 – 12.4)	4.66 (1.37 – 14.9)	0.656	p-p70S6K/p70S6k	0.0789 (0.0312 – 0.3158)	0.1912 (0.0826 – 0.2617)	0.503
STAT3	9.89 (6.71 – 22.9)	18.3 (4.65 – 26.5)	0.824	p-STAT3/STAT3	0.0143 (0.0002 – 0.0855)	0.0821 (0.0343 – 0.3226)	0.261
STAT5	11.8 (11.4 – 22.2)	18.9 (6.84 – 19.5)	0.456	p-STAT5/STAT5	0.0050 (0.0005 – 0.0291)	0.0125 (0.0071 – 0.0361)	0.370

Table S5. Percentages of B cell Subpopulations.

	healthy controls (n=19)			incubation phase (n=7)			early acute infection (n=11)			late acute infection (n=15)			ANOVA	ANCOVA
among gated $CD19^{+}CD45RA^{+}$ lymphocytes [%]	mean	±	SEM	mean	±	SEM	mean	±	SEM	mean	±	SEM	p-value	p-value
age	60.00	±	4.9	61.00	±	6.5	64.73	±	4.1	41.43	±	4.6	0.01	–
activated naive	47.95	±	3.8	56.56	±	2.1	48.63	±	6.9	45.97	±	3.7	0.571	0.561
early memory	11.26	±	1.3	8.25	±	1.2	6.76	±	0.8	12.82	±	1.1	**6.50E-03**	**0.035**
late memory	17.63	±	2.4	10.70	±	1.4	10.07	±	2.3	13.60	±	1.9	0.085	0.09
plasmablasts	1.10	±	0.2	2.88	±	0.8	11.47	±	2.6	4.43	±	1.1	**6.00E-06**	**5.00E-06**
transitionals	7.07	±	0.9	10.45	±	1.3	6.01	±	1.2	7.48	±	1.2	0.151	0.169
virgin naive	13.42	±	1.5	9.43	±	0.9	14.98	±	4.3	12.92	±	1.4	0.577	0.586

virgin naïve: $CD27^{-}CD38^{-}$; activated naïve: $CD27^{-}CD38^{+}$; transitionals: $CD27^{-}CD38^{bright}$; late memory: $CD27^{+}CD38^{-}$; early memory: $CD27^{+}CD38^{dim}$, plasmablasts: $CD27^{+}CD38^{very\ bright.}$

Table S6. Serum Antibodies.

Analyte	healthy controls (n=19) mean ± SEM	incubation phase (n=7) mean ± SEM	early acute infection (n=11) mean ± SEM	late acute infection (n=15) mean ± SEM	ANOVA p-value	ANCOVA p-value
Anti-S1_IgM*	171.76 ± 131.5	131.46 ± 65.4	4735.23 ± 1193.8	5390.80 ± 962.2	**1.5269E-07**	**1.53E-07**
Anti-S1_IgG*	18.17 ± 2.1	19.89 ± 2.5	6100.32 ± 2078.9	9863.68 ± 1569.5	**2.9686E-07**	**7.23E-09**
Anti-S2_IgM*	443.58 ± 167.7	316.86 ± 96.9	3979.75 ± 898.7	4161.82 ± 726.6	**2.0E-06**	**3.93E-07**
Anti-S2_IgG*	1541.97 ± 539.1	1227.86 ± 406.5	15312.57 ± 1667.5	19179.20 ± 1569.7	**3.3328E-16**	**1.37E-16**
Anti-RBD_IgM*	1383.63 ± 453.2	1190.46 ± 435.7	8381.55 ± 1496.8	8646.05 ± 1089.8	**3.3099E-08**	**2.59E-09**
Anti-RBD_IgG*	134.55 ± 19.9	180.11 ± 42.3	9625.34 ± 2391.9	14700.11 ± 1288.4	**3.2145E-12**	**2.68E-13**
Anti-N_IgM*	417.88 ± 153.4	238.11 ± 40.0	7109.66 ± 1438.3	4292.18 ± 964.4	**7.4512E-07**	**5.32E-07**
Anti-N_IgG*	289.08 ± 36.8	503.86 ± 149.2	12357.75 ± 1567.5	14931.00 ± 1386.9	**7.3522E-16**	**1.65E-14**
Anti-S_IgA#	0.34 ± 0.0	0.38 ± 0.0	4.72 ± 1.1	6.64 ± 0.7	**2.5591E-10**	**1.24E-10**

*[MFI]; #[A_{450}].

Table S7. Percentages of Monocyte Subpopulations.

	healthy controls (n=19)			incubation phase (n=7)			early acute infection (n=11)			late acute infection (n=15)			ANOVA	ANCOVA
among gated monocytes	mean	±	SEM	mean	±	SEM	mean	±	SEM	mean	±	SEM	p-value	p-value
$CD14^{+}CD16^{-}$	0.43	±	0.1	2.31	±	1.0	3.11	±	1.7	2.19	±	1.2	0.254	0.251
$CD14^{+}CD16^{+}$	70.69	±	2.7	67.06	±	3.1	68.95	±	3.6	73.35	±	2.7	0.596	0.805
$CD14^{dim}CD16^{-}$	13.04	±	1.5	9.56	±	1.5	11.24	±	2.1	12.24	±	1.6	0.616	0.626
$CD14^{+}CD16^{bright}$	11.12	±	1.0	15.29	±	2.5	9.91	±	1.5	7.19	±	1.0	**4.0E-03**	**0.026**

Table S8. Percentages of Granulocyte Subpopulations.

	healthy controls (n=19)	incubation phase (n=7)	early acute infection (n=11)	late acute infection (n=15)	ANOVA	ANCOVA
among gated granulocytes [%]	mean ± SEM	mean ± SEM	mean ± SEM	mean ± SEM	p-value	p-value
CD177⁻CD11b⁺	19.35 ± 4.0	16.32 ± 4.2	16.24 ± 4.5	13.55 ± 2.2	0.696	0.18
CD177⁺CD11b⁺	32.56 ± 4.9	29.77 ± 8.2	33.64 ± 9.0	35.03 ± 6.2	0.97	0.691
CD177⁺CD11b⁻	28.33 ± 5.1	32.27 ± 8.1	28.15 ± 5.6	29.35 ± 3.5	0.969	0.969
CD177⁻CD11b⁻	19.77 ± 3.7	21.66 ± 5.8	21.96 ± 4.8	22.08 ± 4.9	0.976	0.987
CD177⁻CD16⁺	36.57 ± 5.5	31.93 ± 7.9	34.65 ± 8.0	26.76 ± 5.2	0.664	0.536
CD177⁺CD16⁺	60.01 ± 5.3	63.46 ± 9.1	63.35 ± 8.0	65.68 ± 5.8	0.938	0.558
CD177⁺CD16⁻	0.04 ± 0.0	0.17 ± 0.1	0.29 ± 0.1	0.45 ± 0.1	**0.02**	**0.012**
CD177⁻CD16⁻	2.98 ± 1.5	4.43 ± 3.7	1.72 ± 0.6	7.12 ± 4.6	0.42	0.782

Table S9. Percentages of CD8+ T Cell Subpopulations.

	healthy controls (n=19)			incubation phase (n=7)			early acute infection (n=11)			late acute infection (n=15)			ANOVA	ANCOVA
among CD8+ [%]	mean	±	SEM	mean	±	SEM	mean	±	SEM	mean	±	SEM	p-value	p-value
PD-1−CD27+	58.14	±	6.1	55.16	±	8.1	47.13	±	7.2	66.63	±	5.4	0.237	0.799
PD-1+CD27+	3.97	±	1.8	1.29	±	0.5	13.19	±	3.6	7.84	±	3.2	**0.049**	**0.048**
PD-1+CD27−	3.87	±	1.8	0.86	±	0.6	14.51	±	4.7	5.02	±	2.4	**0.021**	**0.031**
PD-1−CD27−	34.02	±	5.7	42.70	±	8.5	25.17	±	6.7	20.52	±	3.8	0.101	0.23
PD-1+	7.84	±	3.5	2.14	±	0.8	27.70	±	7.6	12.86	±	5.6	**0.023**	**0.027**
CD127−CD27+	11.81	±	1.6	12.07	±	2.4	14.17	±	3.6	21.37	±	2.4	0.178	**0.003**
CD127+CD27+	51.54	±	5.6	46.51	±	8.4	48.34	±	6.5	54.48	±	3.8	0.645	0.816
CD127+CD27−	12.91	±	2.6	11.86	±	3.3	11.43	±	2.3	5.55	±	1.4	0.207	0.598
CD127−CD27−	23.75	±	4.9	29.55	±	7.8	26.06	±	6.7	18.60	±	3.6	0.491	0.941
CD38−CD27+	56.32	±	4.7	47.11	±	8.0	45.28	±	3.9	54.90	±	3.7	0.315	0.14
CD38+CD27+	7.23	±	1.2	11.63	±	1.8	15.15	±	3.5	21.08	±	3.4	**1.39E-03**	**0.002**
CD38+CD27−	6.35	±	1.3	26.41	±	6.5	14.09	±	1.3	5.44	±	0.8	**2.73E-07**	**6.31E-07**
CD38−CD27−	30.10	±	4.8	14.88	±	4.2	25.49	±	6.0	18.58	±	3.2	0.138	0.211

Table S10. Expression of CD38 among CD8+ T Cell Subpopulations.

	healthy controls (n=19)			incubation phase (n=7)			early acute infection (n=11)			late acute infection (n=15)			ANOVA	ANCOVA
CD38 Expression [MFI]	mean	±	SEM	mean	±	SEM	mean	±	SEM	mean	±	SEM	p-value	p-value
$CD8^+$	2038.3	±	150.5	4304.9	±	697.8	3739.0	±	467.9	3847.7	±	398.5	**1.72E-04**	**5.5E-05**
$CD38^+CD27^-$	11940.0	±	540.8	16399.7	±	1172.6	21154.0	±	6389.5	14559.1	±	1684.6	0.150	0.187
$CD27^+CD45RA^+$	17.13	±	4.8	19.34	±	6.0	18.37	±	3.6	33.05	±	4.1	0.052	0.674
$CD27^+CD45RA^-$	17.00	±	2.4	12.77	±	2.3	12.33	±	2.2	10.38	±	1.5	0.131	0.216
$CD27^-CD45RA^-$	5.99	±	1.2	5.28	±	1.6	2.96	±	0.4	2.26	±	0.3	**0.016**	**0.043**
$CD27^-CD45RA^+$	29.74	±	5.5	35.77	±	7.8	35.02	±	6.1	22.24	±	3.6	0.342	0.915
$CD25^-CD127^+$	54.58	±	4.9	46.59	±	6.3	45.05	±	5.2	54.20	±	4.1	0.679	0.293
$CD25^+CD127^+$	7.42	±	0.8	8.06	±	1.5	13.87	±	6.2	6.92	±	2.1	0.546	0.665
$CD25^+CD127^-$	0.88	±	0.2	2.49	±	0.4	2.49	±	0.4	2.28	±	0.4	0.115	0.066
$CD25^-CD127^-$	37.11	±	5.0	42.87	±	7.5	38.59	±	6.6	36.61	±	4.0	0.908	0.35

Table S11. Percantages of CD4+ T Cell Subpopulations.

	healthy controls (n=19)	incubation phase (n=7)	early acute infection (n=11)	late acute infection (n=15)	ANOVA	ANCOVA

among $CD4^+CD45RA^+$ [%]	mean ± SEM	mean ± SEM	mean ± SEM	mean ± SEM	p-value	p-value
$CD27^-CD127^+$	9.08 ± 2.49	4.55 ± 1.95	7.12 ± 3.98	6.10 ± 2.40	0.746	0.765
$CD27^+CD127^+$	72.56 ± 4.13	74.00 ± 4.02	78.70 ± 7.05	74.60 ± 3.49	0.83	0.72
$CD27^+CD127^+CD38^+$	78.99 ± 3.26	78.86 ± 2.12	80.63 ± 2.25	78.69 ± 2.61	0.972	0.77
$CD27^+CD127^+CD38^-$	20.95 ± 3.24	21.10 ± 2.11	19.33 ± 2.25	21.26 ± 2.61	0.972	0.77
$CD27^+CD127^-$	11.09 ± 1.43	17.47 ± 2.94	9.23 ± 1.70	16.13 ± 1.73	**0.012**	**0.042**
$CD27^-CD127^-$	7.27 ± 2.30	3.96 ± 1.31	4.93 ± 3.38	3.18 ± 1.76	0.586	0.732
among $CD4^+CD45RA^-$ [%]						
$CD27^-CD127^+$	16.92 ± 2.18	13.89 ± 4.24	10.71 ± 1.54	11.08 ± 0.83	0.091	0.114
$CD27^+CD127^+$	63.43 ± 2.02	63.89 ± 5.84	72.18 ± 3.52	66.45 ± 1.63	0.135	0.125
$CD27^+CD127^-$	15.18 ± 1.29	18.43 ± 2.48	14.89 ± 2.13	20.29 ± 1.32	0.054	0.147
$CD27^-CD127^-$	4.48 ± 1.01	3.77 ± 2.06	2.22 ± 0.76	2.20 ± 0.50	0.238	0.183
$CD27^+CD38^-$	69.86 ± 2.2	71.49 ± 5.6	73.38 ± 1.8	66.05 ± 2.5	0.288	0.057
$CD27^-CD38^-$	19.47 ± 2.5	14.52 ± 5.0	11.31 ± 1.9	11.38 ± 0.9	**0.035**	0.059
$CD25^+CD127^-$ Tregs	8.95 ± 0.6	11.43 ± 0.8	9.47 ± 1.2	9.76 ± 0.5	0.257	0.242

Table S12. Percantages of NK Cell Subpopulations.

	healthy controls (n=19)	incubation phase (n=7)	early acute infection (n=11)	late acute infection (n=15)	ANOVA	ANCOVA

among gated lymphocytes [%]	mean	±	SEM	mean	±	SEM	mean	±	SEM	mean	±	SEM	p-value	p-value
CD56$^-$CD3$^+$	59.36	±	1.9	49.24	±	3.9	48.88	±	3.9	53.57	±	2.6	0.031	0.014
CD56$^+$CD3$^+$	5.88	±	1.1	3.97	±	1.7	5.01	±	2.1	5.79	±	1.5	0.865	0.89
CD56$^+$CD3$^-$	10.31	±	1.1	13.61	±	2.6	10.60	±	1.1	9.22	±	0.9	0.215	0.38

Table S13. Serum Cytokines.

	healthy controls (n=19)	incubation phase (n=7)	early acute infection (n=11)	late acute infection (n=15)	ANOVA	ANCOVA
Analyte [pg/ml]	mean ± SEM	mean ± SEM	mean ± SEM	mean ± SEM	p-value	p-value
IL-1β	18.18 ± 15.6	2.09 ± 0.0	99.14 ± 49.7	33.23 ± 20.3	0.112	0.109
IL-6	9.81 ± 7.5	21.20 ± 11.7	102.84 ± 48.6	47.02 ± 25.8	0.079	0.045
TNFα	12.76 ± 11.1	1.44 ± 0.0	30.46 ± 18.1	19.43 ± 10.6	0.591	0.566
IP-10	91.13 ± 14.8	402.04 ± 104.2	423.90 ± 75.4	157.12 ± 74.8	**3.97E-04**	**6.12E-04**
IFNλ1	8.67 ± 2.1	5.82 ± 1.0	19.93 ± 8.6	13.52 ± 3.8	0.224	0.239
IL-8	5.66 ± 2.5	12.15 ± 5.5	42.84 ± 20.4	12.12 ± 6.5	0.048	0.05
IL-12p70	2.15 ± 1.4	0.67 ± 0.0	21.23 ± 12.1	6.47 ± 4.4	0.092	0.05
IFNα2	8.34 ± 5.4	12.01 ± 4.9	75.63 ± 43.8	28.07 ± 16.9	0.137	0.074
IFNλ2/3	55.07 ± 28.8	11.13 ± 0.0	79.37 ± 44.1	110.40 ± 57.2	0.54	0.367
GM-CSF	5.12 ± 4.0	1.14 ± 0.0	134.71 ± 82.9	32.68 ± 26.2	0.084	**0.046**
IFNβ	4.41 ± 0.0	5.66 ± 1.2	129.01 ± 84.3	37.34 ± 32.9	0.143	0.057
IL-10	3.98 ± 1.9	3.45 ± 1.2	45.92 ± 23.9	13.37 ± 7.1	0.042	0.032
IFNγ	18.41 ± 17.1	11.93 ± 7.0	70.25 ± 40.3	27.50 ± 20.7	0.409	0.466

Table S14. Absolute Numbers of Live Leukocytes.

	healthy controls (n=19)	incubation phase (n=7)	early acute infection (n=11)	late acute infection (n=15)	ANOVA	ANCOVA
cell count/μL	mean ± SEM	mean ± SEM	mean ± SEM	mean ± SEM	p-value	p-value
granulocytes	2241 ± 279	2025 ± 504	2256 ± 377	2739 ± 709	0.799	0.407
lymphocytes	1788 ± 137	1275 ± 145	943 ± 116	1493 ± 128	**6.3E-04**	**9.4E-04**
CD3	1195 ± 97	740 ± 87	543 ± 82	919 ± 88	**1.02E-04**	**1.04E-04**
CD8	357 ± 43	223 ± 42	132 ± 39	293 ± 46	**6,4E-03**	**9.8E-03**
CD4	693 ± 47	418 ± 46	344 ± 53	531 ± 52	**8.3E-05**	**3.7E-05**
CD19	302 ± 61	103 ± 25	160 ± 28	205 ± 35	0.066	0.074
CD56	205 ± 25	159 ± 39	106 ± 18	136 ± 17	**0.030**	**0.038**
monocytes	388 ± 29	361 ± 36	414 ± 78	368 ± 60	0.923	0.859

Table S15. Apoptotic among Live Leukocytes.

	healthy controls (n=19)			incubation phase (n=7)			early acute infection (n=11)			late acute infection (n=15)			ANOVA	ANCOVA
[%]	mean	±	SEM	mean	±	SEM	mean	±	SEM	mean	±	SEM	p-value	p-value
granulocytes	3.14	±	2.56	22.93	±	22.71	2.51	±	1.72	5.21	±	3.66	0.266	0.217
lymphocytes	0.57	±	0.30	0.11	±	0.03	3.48	±	2.12	0.48	±	0.18	0.078	0.117
CD3	0.75	±	0.41	0.14	±	0.05	5.33	±	3.02	0.81	±	0.36	0.05	0.076
CD8	0.11	±	0.05	0.03	±	0.01	2.90	±	2.25	0.51	±	0.30	0.17	0.219
CD4	0.06	±	0.02	0.02	±	0.01	1.65	±	1.45	0.25	±	0.16	0.252	0.314
CD19	4.19	±	3.62	0.22	±	0.08	5.96	±	4.33	0.91	±	0.46	0.625	0.744
CD56	0.22	±	0.06	0.08	±	0.01	2.12	±	1.60	0.21	±	0.06	0.153	0.221
monocytes	0.45	±	0.11	0.32	±	0.14	6.83	±	5.66	0.75	±	0.34	0.214	0.228

Table S16. Correlations among Humoral and Cellular Immune Parameters in Healthy Controls.

	monocytes $CD14^+CD16^{bright}$	granulocytes $CD177^+CD16^-$	serum conc. IP-10	plasmablasts	anti-S1 IgM	anti-S1 IgG	anti-S IgA	cytotoxic T $CD38^+CD27^-$	cytotoxic T PD-1+	mfi CD38 on cytotoxic T
monocytes $CD14^+CD16^{bright}$	1									
granulocytes $CD177^+CD16^-$		1								
IP-10 serum conc.	r = 0.746<br>p = 0.001		1							
plasmablasts				1						
anti-S1 IgM					1					
anti-S1 IgG						1				
anti-S1 IgA							1			
cytotoxic T $CD38^+CD27^-$								1		
cytotoxic T cells $PD-1^+$									1	
MFI CD38 on cytotoxic T cells										1

Spearman rank correlation analysis. The table only contains results that withstand Bonferroni correction.

Table S17. Correlations among Humoral and Cellular Immune Parameters in COVID-19 Patients.

	monocytes $CD14^{+}CD16^{bright}$	granulocytes $CD177^{+}CD16^{-}$	serum conc. IP-10	plasmablasts	anti-S1 IgM	anti-S1 IgG	anti-S IgA	cytotoxic T $CD38^{+}CD27^{-}$	cytotoxic T PD-1+	mfi CD38 on cytotoxic T
monocytes $CD14^{+}CD16^{bright}$	1									
granulocytes $CD177^{+}CD16^{-}$		1								
IP-10 serum conc.			1							
plasmablasts				1						
anti-S1 IgM		r=0.594 p<0.0001			1					
anti-S1 IgG		r=0.594 p<0.0001			r=0.810 p< 0.0001	1				
anti-S1 IgA		r=0.609 p<0.0001			r=0.882 p< 0.0001	r=0.926 p< 0.0001	1			
cytotoxic T cells $CD38^{+}/CD27^{-}$			r=0.667 p<0.0001					1		
cytotoxic T cells $PD\text{-}1^{+}$									1	
MFI CD38 on cytotoxic T cells										1
